# Multi‐Regional Characterization of Moroccan Bee Pollen: Nutritional, Bioactive, and Functional Properties

**DOI:** 10.1002/fsn3.72174

**Published:** 2026-10-07

**Authors:** Fedoua Diai, Toufik Bouddine, Mohamed Reda Kachmar, Morad Kaddouri, Charaf Eddine Kassimi, Ibtissame Guirrou, Hassan Laaroussi, Mohammed Al‐zharani, Fahd A. Nasr, Ashraf Ahmed Qurtam, Lhoussain Hajji, Said Chakir

**Affiliations:** ^1^ Human Nutrition, Bioactives and Oncogenetics Team, Faculty of Science Moulay Ismail University Meknes Morocco; ^2^ High Institute of Nursing Professions and Health Techniques Beni Mellal Morocco; ^3^ Laboratory of the Engineering and Applied Technologies, Higher School of Technology Sultan Moulay Slimane University Beni Mellal Morocco; ^4^ Argo‐Food Technology and Quality Laboratory Regional Center of Agricultural Research of Meknes Rabat Morocco; ^5^ Laboratory of Biotechnology, Conservation and Valorization of Bioresources (BCVB), Research Unit: Api‐Phytotherapy, Physiology, Environment and Health, Department of Biology, Faculty of Sciences Dhar Mehraz Sidi Mohamed Ben Abdellah University Fez Morocco; ^6^ Biology Department, College of Science Imam Mohammad Ibn Saud Islamic University (IMSIU) Riyadh Saudi Arabia

**Keywords:** α‐amylase and α‐glucosidase, bee pollen, botanical origin, phytochemical

## Abstract

Bee Pollen is a natural beehive product consisting of a complex mixture of floral pollen, nectar, honey, and bee salivary enzymes. It has gained increasing attention as a valuable functional food. The aim of this study was to characterize eight Bee Pollen samples collected from different Moroccan regions in terms of physicochemical parameters, nutritional composition, mineral profile, phenolic content, antioxidant capacity, individual phenolic compounds and in vitro antidiabetic potential. The samples exhibited acceptable physicochemical parameters, with acceptable energy values ranging from 156.85 to 205.41 kcal/100 g. Potassium and calcium were the predominant minerals with values of 741.82–2116.99 mg/kg and 676.53–2197.15 mg/kg respectively. The total phenolic content ranged between 4.19 and 17.39 mg GAE/g while the total flavonoid content ranged between 0.94 and 3.18 mg QE/g. Bee Pollen extracts exhibited strong antioxidant activity, with DPPH IC_50_ values of 28–64 μg/mL, ABTS IC_50_ values of 0.12–0.28 mg/mL, and FRAP values of 87.33–146.92 μmol Fe^2+^/g, along with notable α‐amylase and α‐glucosidase inhibitory activities (IC_50_ = 263.40–953.66 and 145.50–670.26 μg/mL, respectively). HPLC analysis revealed the presence of several bioactive phenolic compounds. Our results can indicate Moroccan Bee Pollen as a nutrient and phenolic rich natural product with promising antioxidant and antidiabetic potential for functional food applications.

## Graphical abstract

This study characterized eight Moroccan bee pollen samples from different regions for their nutritional composition, mineral profile, phenolic content, antioxidant capacity, and antidiabetic potential. Results revealed significant regional variability, with potassium and calcium as dominant minerals, and strong antioxidant and α‐amylase/α‐glucosidase inhibitory activities, highlighting Moroccan bee pollen as a promising functional food ingredient.

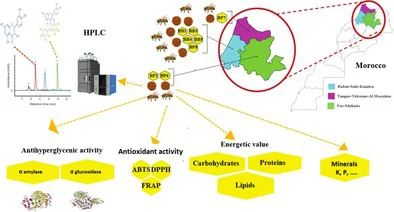

## 1 Introduction

Bee pollen (BP) is increasingly consumed as a dietary supplement and incorporated into functional foods because it contains high‐quality proteins, essential amino acids, unsaturated fatty acids, vitamins, minerals, and diverse phenolic compounds (Almeida‐Muradian et al. 2005; Campos et al. 2008) Numerous studies have demonstrated that BP exhibits antioxidant, anti‐inflammatory, antimicrobial, and immunomodulatory properties, supporting its potential role in managing oxidative stress and metabolic disorders (Pascoal et al. 2014; Aylanc et al. 2023). The biochemical composition, functional properties, and health‐promoting potential of BP are, however, highly dependent on its botanical and geographical origin, climatic conditions, and processing or storage practices (Thakur and Nanda 2020; Campos et al. 2008), which complicates compositional standardization and the substantiation of health‐related claims.

In Morocco, apicultural products hold growing economic and nutritional importance, yet research on Moroccan BP remains limited and geographically fragmented. Previous investigations have mainly addressed physicochemical or antioxidant parameters in isolation, rarely integrating compositional and functional attributes or providing cross‐regional comparisons (Aylanc et al. 2023; Laaroussi et al. 2023). Data on mineral profiles, heavy‐metal contamination, and bioactivity relationships also remain scarce. Furthermore, the association between floral origin and phenolic composition is frequently hypothesized but rarely confirmed by melissopalynological analysis (Aylanc et al. 2023; Campos et al. 2008).

Accordingly, this study aimed to provide an integrated characterization of Moroccan BP collected from eight regions. The objectives were to determine its physicochemical and nutritional composition, quantify mineral elements including potential contaminants, identify major phenolic compounds, and evaluate in vitro antioxidant and α‐amylase/α‐glucosidase inhibitory activities. We hypothesized that the botanical and geographical origin of Moroccan BP significantly influences its biochemical composition and biological activities, and that an integrated multi‐regional approach would reveal meaningful compositional patterns relevant to its valorization as a functional food ingredient.

## 2 Materials and Methods

### 2.1 Chemicals

Ethanol (70%); aluminum chloride (AlCl_3_); Folin–Ciocalteu reagent; gallic acid; quercetin; 2,2‐diphenyl‐1‐picrylhydrazyl radical (DPPH); ferrous sulfate (FeSO_4_); sodium nitrite (NaNO_2_); sodium sulfate (Na_2_SO_4_); sodium carbonate (Na_2_CO_3_); sodium hydroxide (NaOH); Bradford reagent; bovine serum albumin (BSA); glucose; disodium hydrogen phosphate (Na_2_HPO_4_); potassium dihydrogen phosphate monohydrate (KH_2_PO_4_·H_2_O); potassium ferricyanide (K_3_Fe(CN)_6_); trichloroacetic acid (TCA); ferric chloride hexahydrate (FeCl_3_·6H_2_O); nitric acid (HNO_3_); potassium persulfate (K_2_S_2_O_8_); ABTS; methanol; petroleum ether. Chemicals employed in this work were of analytical grade and Ultrapure water was obtained by Ultra‐pure water system (SG Ver. 1.11. Water LLC, USA).

### 2.2 Collection of Bee Pollen Samples

In the present study, a preliminary set of 8 BP samples was acquired using a convenience sampling approach to assess variability directly from hygienic beehives equipped with pollen traps situated in various regions of Morocco (Figure 1) during March and April 2023. This sample size provides initial characterization data but is recognized as insufficient for comprehensive geographical or botanical profiling which would require a larger systematically designed survey. To ensure the quality of the samples, they were preserved in food‐grade jars and stored at −20°C immediately after collection to preserve their quality until use. Detailed information about flowering source and the geographical coordinates of beehives are presented in (Table 1).

**FIGURE 1:**
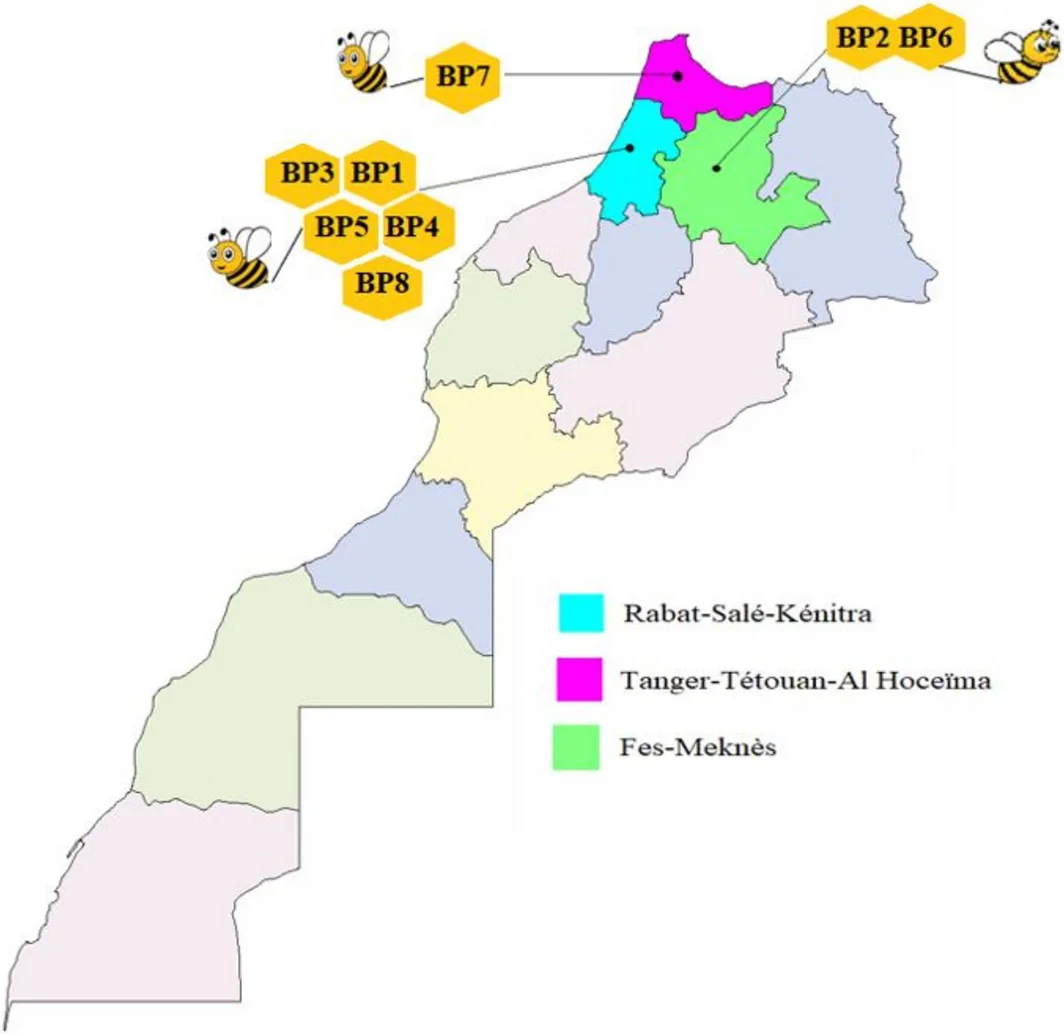
Geographical location of bee pollen samples.

**TABLE 1:** BP Geographical location, harvest period and predominant plant species.

Samples	Region	Locality name	Latitude (N)	Longitude (W)	Altitude (m)	Harvest period	Surrounding plant species
BP1	Rabat‐Salé‐Kénitra	Jorf el malha	34°28′60″	5°31′00″	35	March 2023	*Petroselinum crispum* , *Citrus sinensus L*.
BP2	Fes‐Meknès	Sebt jahjouh	33°42′59″	5°40′22″	719	May 2023	*Pimpinella anisum* , *Calendula officinalis* , *Sinapis arvensis*
BP3	Rabat‐Salé‐Kénitra	Sidi kacem	34°13′00″	5°42′00″	194	May 2023	*Citrus sinensus L., Calendula officinalis, Petroselinum crispum*
BP4	Rabat‐Salé‐Kénitra	Tiflet	33°53′35″	6°18′25″	35	April 2023	*Papaver rhoeas L*., *Calendula officinalis*, *Sinapis arvensis*
BP5	Rabat‐Salé‐Kénitra	Sidi slimane	34°15′35″	5°55′45″	41	April 2023	*Eucalyptus globulus* , *Brassica napus L*., *Sinapis arvensis*
BP6	Fes‐Meknès	Boufekrane	33°45′26″	5°29′07″	726	April 2023	*Papaver rhoeas L*., *Calendula officinalis*, *Malus domestica* , *Pirus communis L*.
BP7	Tanger‐Tétouan‐Al Hoceïma	Ouazzane	34° 47′50″	5°34′56″	614	May 2023	*Nigelle sativa*, *Citrus sinensus L*., *Sinapis arvensis, Papaver rhoeas L*.
BP8	Rabat‐Salé‐Kénitra	Douar lafdoul	34° 32′4″	5°50′41″	59	April 2023	*Citrus sinensus L*., *Papaver rhoeas L*., *Petroselinum crispum*, *Sinapis arvensis L*., *Calendula officinalis*

### 2.3 Extraction of Bee Pollen Samples

1 g of each BP sample was weighed and extracted by maceration with 30 mL of ethanol (70% v/v) under mechanical shaking (170 rpm) for 1 week in the dark at room temperature. The mixtures were then sonicated for 30 min in an ultrasonic bath (Biobase ultrasonic cleaner UC‐VAD, Shandong, China) and filtered through Whatman No. 1 filter paper.

### 2.4 Determination of Botanical Origin

The palynological characterization of the BP samples was carried out following the methodology described by Almeida et al. (2017). Briefly, 2 g of each sample, corresponding to approximately 350 pollen pellets, was considered representative of the botanical origine. The pollen loads of each sample were grouped into subsamples according to color, and each subsample was weighed to calculate its percentage in the main sample. According to the classification proposed by Louveaux et al. (1978), pollen types were categorized as predominant pollen (≥ 45%), secondary pollen (16%–44%), important minor pollen (3%–15%), and minor pollen (< 3%). Scanning electron microscopy (SEM) analysis was carried out using a QUATTRO S‐FEG microscope (Thermo Fisher Scientific, Waltham, MA, USA) (Figure 2).

**FIGURE 2:**
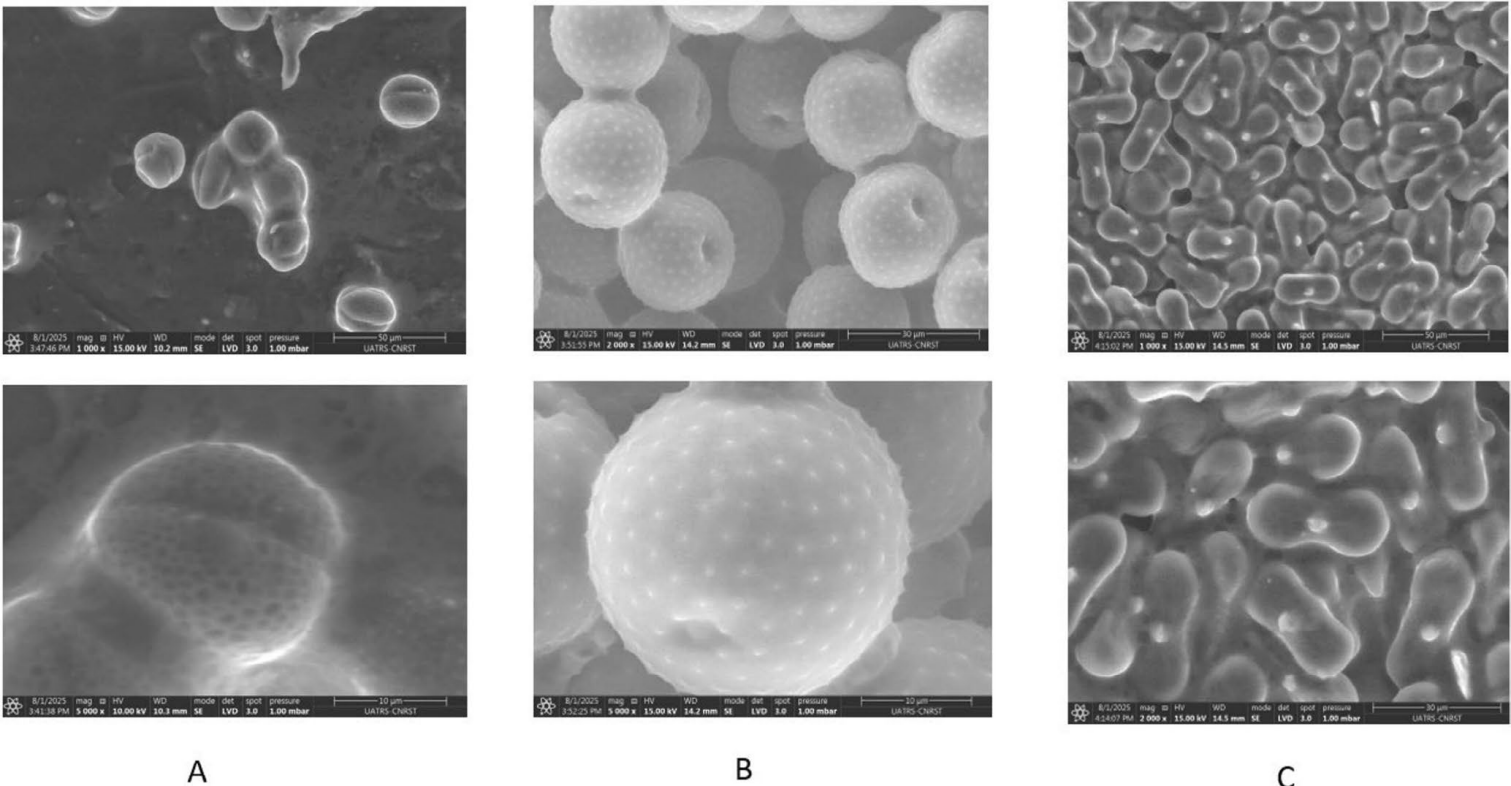
Scanning Electron Microscope (SEM) micrographs of some of the observed pollen types in the Bee Pollen samples. (A) Brassicaceae; (B) Asteraceae; (C) Apiaceae.

### 2.5 Physicochemical Analyses of Bee Pollens

#### 2.5.1 Moisture Content (%)

Moisture content was determined using the oven‐drying method to constant weight. Approximately 3 g of each sample was accurately weighed and dried in an oven at 65°C for 24 h. Moisture content was calculated using Equation (1):

(1) $$\text{Moisture Content}\;\left(\%\right)=\frac{\left(\mathrm{WS}-\mathrm{WD}\right)}{\mathrm{WD}}\times 100$$

where WS is the weight of the sample and WD is the weight of the dried sample.

#### 2.5.2 Water Activity (A_w_)

The water activity of BP samples was determined using a Rotronic water activity meter HP23‐AW‐A (Rotronic AG, Bassersdorf, Switzerland).

#### 2.5.3 pH

The pH of the BP samples was measured with a pH meter (J.P. Selecta S.A., Abrera, Barcelona, Spain). For this purpose, 5 g of each sample was dispersed in 20 mL of distilled water and measurements were taken at room temperature after equilibration.

#### 2.5.4 Ash

Ash content was determined gravimetrically to constant weight. 2 g of each sample were weighed into porcelain crucibles pre‐incinerated on a hotplate and then incinerated in a muffle furnace at 550°C for 6 h (Costa et al. 2017).

#### 2.5.5 Minerals

Mineral elements were determined by the calcination method using inductively coupled plasma–atomic emission spectroscopy ICP‐AES following the procedure described by Laaroussi et al. (2023). The resultant ash was moistened with 5 mL of 0.1 M nitric acid and evaporated on a heating plate to near dryness, then dissolved in 10 mL of the same acid and made up to 25 mL with distilled water. Calcium (Ca), potassium (K), sodium (Na), zinc (Zn), and magnesium (Mg) were quantified using ICP‐AES after appropriate calibration with standard solutions. All determinations were performed in triplicate.

### 2.6 Bioactive Compounds and Chemical Composition of Bee Pollen

#### 2.6.1 Total Phenolic Content (TPC)

The total phenolic content of the pollen extract was estimated using the Folin–Ciocalteu method which is based on the colorimetric reduction/oxidation reaction of phenols as described by Ferreira‐Santos et al. (2020). Briefly, 50 μL of each ethanolic extracts was mixed with 250 μL of Folin‐Ciocalteu reagent (0.2 N) and 200 μL of Na_2_CO_3_ (7.5%). The absorbance was measured at 760 nm after 2 h of incubation. Gallic acid was used as a standard to achieve the calibration curve (R2 = 0.996) with the regression equation: Y=78.314x+0.134 (Figure S1). The results were expressed in mg gallic acid equivalent (GAE) per gram of BP (mg GAE/g of BP) (Figure S1).

#### 2.6.2 Total Flavonoid Content (%) (TFC)

The total flavonoid content was determined following the colorimetric method described by Ferreira‐Santos et al. (2020). A mixture of 200 μL of Al_2_Cl_3_ (2%), 50 μL of ethanolic pollen extract or standard was incubated for 1 h. The absorbance was measured at 420 nm with a (Jasco V‐730 spectrophotometer, Tokyo, Japon). Quercetin was used as a standard to achieve the calibration curve (R2 = 1) with the following regression equation: y=56.161x+0.1248 (Figure S2). The results were expressed in milligram of quercetin equivalent (Quercetin Equivalent) per gram of pollen (mg QE/g). Tests were carried out in triplicate (Figure S2).

#### 2.6.3 Phenolic Compounds Identification

Phenolic compounds were separated on a Nucleosil C18 column (5 μm. 250 × 4.6 mm; Macherey‐Nagel) with UV detection at 280 nm (Jasco ChromNAV 2.0). Mobile phase: 0.01% phosphoric acid (A) and acetonitrile (B); gradient (% B): 5 (0 min). 15 (3). 25 (13). 30 (25). 35 (35). 45 (39). 55 (47). 75 (56). 100 (60–65). 5 (73–80 min). Flow: 0.9 mL/min; injection: 20 μL; temperature: 25°C (Kachmar et al. 2019). Compounds were identified qualitatively by comparing retention times with authentic standards. Limits of detection (LOD) and quantification (LOQ) were not determined as this analysis was performed for identification purposes only, not quantitative analysis.

### 2.7 Antioxidant Activity

The antioxidant activity of ethanolic BP extracts was determined using three methods:

### 2.8 DPPH (Free‐Radical Scavenging Activity)

The antioxidant activity of each pollen sample was determined by the DPPH radical scavenging activity assay described by Bouddine et al. (2022). 50 μL of ethanolic extract was added to 950 μL of ethanolic solution of DPPH (0.025%). After 1h in the dark, the absorbance was measured against the control at 𝜆 = 517 nm using UV visible spectrophotometer.

The percentage of the DPPH inhibition was calculated using the following Equation (2):

(2) $$\%\text{inhibition}=\frac{\left(A\;\text{control}-A\;\text{sample}\right)}{A\;\text{control}}$$

where A, Absorbance.

IC_50_ values were from dose‐response curves.

#### 2.8.1 FRAP (Ferric Reducing Antioxidant Power)

The FRAP assay was conducted following the protocol outlined by Asmae et al. (2021). 100 μL of pollen extract were mixed with 500 μL of phosphate buffer and 500 μL of potassium ferricyanide. After incubation at 50°C for 20 min, 500 μL of trichloroacetic acid (TCA) was added and the mixture was then centrifuged for 10 min at 3000 rpm. Next, 500 μL of distilled water was added to 100 μL of supernatant and 100 μL of FeCl_3_. The absorbance was measured at 700 nm.

#### 2.8.2 ABTS (2.2′‐Azino‐Bis (3‐Ethylbenzothiazoline‐6‐Sulfonic Acid)

The scavenging activity of the ABTS radical cation (ABTS^•+^) was evaluated according to the method described by Ferreira‐Santos et al. (2020). In brief, the (ABTS^•+^) radical was generated by reacting a 7 mM ABTS aqueous solution with 2.45 mM potassium persulfate (K_2_S_2_O_8_) in the dark for 16 h. The absorbance was then adjusted to 0.7 ± 0.05 at 734 nm at room temperature, 10 μL of ethanolic BP extract or distilled water (control) was added to 990 μL of the (ABTS^•+^) solution. Absorbance measurements were taken at 734 nm 5 min after initial mixing using water as the blank. The ABTS‐radical‐cation‐inhibition percent was determined using the following Equation (3):

(3) $${\mathrm{IC}}_{50}\%=\frac{\left(A_{0}-A_{1}\right)}{A_{0}}\times 100$$

where A_0_ is the absorbance of the control and A_1_ is the absorbance of the sample.

IC_50_ from dose–response curves.

### 2.9 In Vitro Digestive Enzymes' Inhibitory Activity

The BP hydro‐ethanolic extracts (ethanol‐distilled water 70%. v/v) action in the inhibition of digestive α‐amylase and α‐glucosidase were determined calorimetrically using UV/Vis spectrophotometer as described previously by Laaroussi et al. (2023) as follows:

#### 2.9.1 α‐Amylase Assay

A volume of 500 μL of α‐amylase solution (0.5 mg/mL) was mixed with 500 μL of each BP extract at different concentrations, while 70% ethanol served as the negative control. All mixtures were then incubated at 37°C for 15 min. Acarbose (Sigma‐Aldrich) at a range of concentrations was used as a positive control for both enzymatic assays. Following incubation with the enzyme, 500 μL of 1% starch solution was introduced into the reaction mixture, which was further incubated at 37°C for 15 min. Thereafter, 1 mL of dinitrosalicylic acid (DNS) reagent was added, and the mixtures were heated in a boiling water bath for 10 min. The resulting mixture was diluted 10 times and the absorbance was read at 540 nm.

#### 2.9.2 α‐Glucosidase Assay

Hydroethanolic bee pollen extracts (BP1–BP8) were incubated with α‐glucosidase (10 U/mL) and p‐nitrophenyl‐α‐D‐glucopyranoside (pNPG, 3 mM) at 37°C for 15 min. The reaction was stopped by adding 1 M Na_2_CO_3_, and the absorbance of the released p‐nitrophenol was measured at 400 nm. The percentage inhibition of α‐amylase and α‐glucosidase was determined using the following Equation (4):

(4) $$\%\text{inhitbition}=\frac{\left(A\;\text{control}-A\;\text{sample}\right)}{\text{Acontrol}}\times 100$$

The pollen concentration required to inhibit 50% (IC_50_) of α‐amylase and α‐glucosidase activities were calculated from a dose–response curve, and the results were expressed in μg/mL.

### 2.10 Energetic Value of Bee Pollen Samples

#### 2.10.1 Carbohydrates

The total soluble carbohydrate (TSC) content was determined via the phenol‐sulfuric acid method as described by Ferreira‐Santos et al. (2020). 50 μL of the extract was mixed with 150 μL of sulfuric acid (H_2_SO_4_. 96‐98% v/v). Then, 30 μL of phenol reagent (5%) was added to the mixture. This solution was then heated at 90°C for 5 min. After allowing the mixture to cool to room temperature for 5 min, the absorbance was measured at 490 nm using a UV/Vis spectrophotometer. Glucose was used as a standard to achieve the calibration curve R2=0.992. The total carbohydrates content was expressed as a milligram of glucose equivalents (GlcE) per g of pollen (mg GLcE/g).

#### 2.10.2 Lipids

The lipid content was measured using a Soxhlet apparatus (Model SER 148/3, VELP SCIENTIFICA, Usmate Velate, Italy). 2 g of each sample were weighed and placed into thimbles (123 × 43 mm i.d.; Whatman International; Brentford, UK). Petroleum ether (120 mL) was then added to each extraction cup. The samples along with the extraction cups, underwent the following processes: boiling for 40 min, rinsing for 20 min and recovery for 10 min (Hii et al. 2023). After completion, the extraction cups containing the residues were weighed. The lipid content (%) was calculated following Equation (5):

(5) $$\text{Lipid content}\left(\%\right)=\frac{\mathrm{WF}-W}{\mathrm{WS}}\times 100$$

where WF, Weight of extraction cup + residue; W, Weight of extraction cup; WS, Weight of sample.

#### 2.10.3 Proteins

##### 2.10.3.1 Soluble Proteins

The soluble protein content was obtained using the Bradford assay. For this, 20 μL of pollen extract was mixed with 230 μL of Bradford dye reagent. The mixture was placed in the dark for 5 min and the absorbance was measured at 595 nm by (Jasco V‐730 spectrophotometer UV/Vis). Bovine albumin serum (BSA) was used to perform the standard curve (R2 = 0.989) and the results were expressed as milligram of BSA equivalents per gram of pollen (mg BSA/g) (Ketkar et al. 2014).

##### 2.10.3.2 Total Proteins

Protein content was estimated using the official Kjeldahl method (AOAC 979.09). Briefly, 0.5 g of sample was digested with 6 mL of concentrated sulfuric acid (H_2_SO_4_) in the presence of a Cu‐Se catalyst (AppliChem, Gatersleben, Germany). The digestion process was performed by heating the flasks at 450°C for 90 min using a DK‐20 digester (VELP Scientifica, Usmate Velate, Italy). The subsequent distillation was carried out in an automatic distillation unit (UDK‐149. VELP Scientifica. Usmate Velate. Italy) using 32% sodium hydroxide and a 3% boric acid solution. Finally, the titration step was performed with 0.01 M HCl in an automatic titrator (TitroLine, VELP Scien‐tifica, Usmate Velate, Italy). The total protein content (%) was calculated using the following Formula (6):

(6) $$\text{Kjeldahl Nitrogen}\;\left(\%\right)=\frac{\left(\mathrm{VS}-\mathrm{VB}\right)\times M\times 14.01}{W\times 10}$$

$$\text{Protein Content}\;\left(\%\right)=\%\text{Kjeldahl Nitrogen}\times 6.25$$

where VS, volume (mL) of standardized acid used to titrate a sample; VB, Volume (mL) of standardized acid used to titrate reagent blank; M, Molarity of standard HCl; 14.01: atomic weight of N; W, weight (g) of sample; 10: factor to convert mg/g to percent; and 6.25: factor to convert N to protein (Ketkar et al. 2014)

### 2.11 Total Energy

The total energy was estimated according to the Atwater system based on the protein, carbohydrate, and lipid contents of the all BP samples (Feas et al. 2012) (7).

(7) $$\text{Total energy}\;\left(\text{kcal}/100\;g\right)=\left(4\times \text{Protein}\right)+\left(9\times \mathrm{Fat}\right)+\left(4\times \text{Carbohydrate}\right)$$

### 2.12 Statistical Analysis

The data are presented as means ± SD. Prior to Principal Component Analysis (PCA), the suitability of the data was assessed. The Kaiser‐Meyer‐Olkin (KMO) measure verified the sampling adequacy for the analysis, with a value of 0.72 which is considered “middling” and above the acceptable threshold of 0.50 (Field 2013). Bartlett's test of sphericity was significant (*χ* ^2^(55) = 328.45. *p* < 0.001) indicating that the correlations between variables were sufficiently large for PCA. The number of components to retain was determined using the Kaiser criterion, which selects components with eigenvalues greater than 1. The retained components collectively explained 68.4% of the total variance in the dataset (Figure S3). Comparisons of treatment means (LSD. 5% level) were conducted using the SPSS 22 statistics software (IBM Software Lab. Rome. Italy).

## 3 Results and Discussion

### 3.1 Botanical Origins of Bee Pollen Samples

Microscopic examination of the samples revealed multiple morphologically distinct pollen types, differing in size, shape, exine ornamentation, and aperture configuration. This diversity confirmed the polyfloral nature of all samples and precluded any monofloral classification (Louveaux et al. 1978). Despite this shared polyfloral character, palynological profiles varied considerably among samples in terms of pollen composition.

Across all samples, a total of 8 pollen families were identified. BP5 was the most botanically restricted, comprising exclusively Myrtaceae (51%) and Brassicaceae (49%), whereas all remaining samples contained at least three families. BP1, BP2, BP3, BP4, and BP6 each yielded three families, while BP7 and BP8 exhibited greater botanical richness, with four and five families, respectively. The full palynological results are reported in (Table 2). Brassicaceae emerged as the most ubiquitous taxon, detected in all samples at varying frequencies with the sole exception of BP6, suggesting that this family constitutes a predominant and widespread floral resource for honeybees across the sampled sites. In contrast, *Myrtaceae* was recorded exclusively in BP5, a pattern likely attributable to localized foraging behavior associated with the proximity of *Eucalyptus* or related taxa. The variability in pollen richness among studied samples may reflect differences in local floral availability and landscape heterogeneity at each collection site (Louveaux et al. 1978). These differences in the relative abundance of pollen at family level may contribute to variations in the phytochemical composition and antioxidant potential of the samples. According to (Bakchiche et al. 2020), BP samples collected from the Laghouat region revealed a diverse botanical composition, encompassing taxa from several plant families, including *Asteraceae, Betulaceae, Boraginaceae, Brassicaceae, Cistaceae, Ericaceae, Fabaceae, Liliaceae, Myrtaceae, Salicaceae*, and *Rosaceae*. Among these, *Brassicaceae* was identified as the dominant family. Similarly, Mokhtari et al. (2022) characterized 8 BP samples collected from different Algerian regions as polyfloral. A total of 40 pollen types distributed among 22 botanical families were identified, among which *Asteraceae* and *Brassicaceae* were the most represented. The predominance of these families may be attributed to the fact that many of their species are recognized as important nectar sources for honeybees (Boulfous et al. 2025).

**TABLE 2:** Palynological analysis of BP samples.

Samples	Families	Relative frequency (%) of pollen types	Classification
BP1	*Apiaceae*, *Rutaceae*, *Brassicaceae*	*Apiaceae* (40%), *Rutaceae* (33%) and *Brassicaceae* (27%)	Polyfloral
BP2	*Apiaceae*, *Asteraceae*, *Brassicaceae*	*Apiaceae* (52%), *Asteraceae* (33%) and *Brassicaceae* (15%)	Polyfloral
BP3	*Rutaceae*, *Apiaceae*	*Rutaceae* (45%), *Brassicaceae* (20%) and *Apiaceae* (35%)	Polyfloral
BP4	*Papaveraceae*, *Asteraceae*, *Brassicaceae*	*Papaveraceae* (45%), *Asteraceae* (28%) and *Brassicaceae* (27%)	Polyfloral
BP5	*Myrtaceae*, *Brassicaceae*	*Myrtaceae (51%), Brassicaceae* (49%)	Polyfloral
BP6	*Papaveraceae*, *Asteraceae*, *Rosaceae*	*Papaveraceae* (47%), *Asteraceae* (30%) and *Rosaceae* (23%)	Polyfloral
BP7	*Ranunculaceae*, *Rutaceae*, *Brassicaceae*, *Papaveraceae*	*Ranunculaceae* (46%), *Rutaceae* (29%), *Brassicaceae* (17%) and *Papaveraceae* (8%)	Polyfloral
BP8	*Rutaceae*, *Papaveraceae*, *Apiaceae*, *Brassicaceae*, *Asteraceae*	*Rutaceae* (48%), *Papaveraceae* (24%), *Apiaceae* (12%), *Brassicaceae* (10%) and *Asteraceae* (6%)	Polyfloral

### 3.2 Moisture

Moisture content is one of the major quality factors affecting the shelf time life of BP (Thakur and Nanda 2020). The moisture content of the analyzed samples varied from 3.22% ± 0.05% for BP1 harvested from Jerf Elmalha to 27.48% ± 0.017% for BP3 harvested in Sidi Kassem with an average of 16.26%. Lower values were reported by Asmae et al. (2021) in fresh BP harvested from different regions in Morocco (10.7%–26.8% with an average of 20.29%). This is probably attributed to the harvesting and storage conditions (Aylanc et al. 2021). Similar results were found in BP samples collected from Bulgaria with an average of 17.3% (Radev 2018), Colombia, Italy and Spain with an average of 15% (Gardana et al. 2018) while (Laaroussi et al. 2023) reported moisture levels of 3.16%–14.08% with a mean value of 5.09% was reported for Moroccan dry BP. As reported by Campos et al. (2008), fresh BP should contain between 10% and 30% of water which is in accordance with the results observed in our study. Excepted BP1 and BP2, this is probably attributed to the harvesting and/or storage condition. Fresh BP generally retains a higher nutritional value than dried BP, whose moisture content is typically reduced to below 8%. Nevertheless, the high moisture content of fresh BP creates an ideal environment for the growth of diverse micro‐organisms particularly yeasts and molds. To maintain the quality of BP, it is recommended that it is harvested daily from pollen traps and stored in a deep freezer at temperatures below −18°C until consumed (Radev 2018).

### 3.3 Water Activity (A_w_)

Water activity measures the amount of available water for spoilage microorganisms like Bacteria, Molds and yeast. It's a critical factor affecting the shelf life of collected BP (Anjos et al. 2019). A higher water activity promotes the growth of microorganisms, particularly yeasts and molds which can produce mycotoxins and ochratoxins posing significant health risks to consumers (Pascoal et al. 2023). The water activity values obtained in this study was higher in BP7 and BP8 (0.41) and lower in BP2 (0.31). These results fall within the range reported by Pascoal et al. (2023) for BP harvested in Northeast Portugal (from 0.36 to 0.41) and by Oroian et al. (2022) for BP collected from the Northeast Romania (from 0.17 to 0.55). Anjos et al. (2019) reported higher water activity in frozen BP (from 0.58 to 0.72) compared to the dry one (from 0.26 to 0.34). The variation in water activity among the samples is attributed to differences in botanical origin and storage conditions. Additionally, our findings suggest that the water activity of the examined BP samples is sufficiently low to inhibit the growth of yeasts, molds and bacteria which typically require water activity levels above 0.61 and 0.91, respectively.

### 3.4 pH

The *p*H values of our samples ranged from 4.90 ± 0.009 in BP1 to 5.13 ± 0.04 in BP4 with an average of 4.99 (Table 3). These results are in agreement with those reported by Aylanc et al. (2023), which averaged around 4.7, similar pH values were obtained for BP collected from different areas in Morocco by Asmae et al. (2021) with pH values ranging from 4.19 to 4.8. Similar values were described by Thakur and Nanda (2018), who reported pH values varying between 4.74 and 5.48. These results align with the pH range specified by Brazilian legislation, which extends from 4 to 6 (Costa et al. 2017). According to the literature, a pH value below 4 can promote the growth of undesirable microorganisms in pollen (Anjos et al. 2023) causing problems with texture and stability and therefore shelf life of BP (Straumite et al. 2022).

**TABLE 3:** Moisture content, pH, water activity (Aw), and ash content of BP samples.

BP	Moisture (%)	pH	Aw	Ash (%)
BP1	5.77 ± 0.02ᶜ	4.95 ± 0.05ᵇ	0.394 ± 0.001ᵇᶜ	3.72 ± 0.03ᶜ
BP2	3.22 ± 0.05ᵃ	4.93 ± 0.01ᵇ	0.316 ± 0.001ᵃ	4.64 ± 0.03ᵈ
BP3	27.48 ± 0.02ᵉ	4.92 ± 0.03ᵇ	0.401 ± 0.001ᶜ	3.98 ± 0.03ᶜ
BP4	14.87 ± 0.03ᵈ	5.13 ± 0.04ᵃ	0.333 ± 0.001ᵃ	4.06 ± 0.03ᶜ
BP5	16.03 ± 0.02ᵈ	5.06 ± 0.01ᵃᵇ	0.343 ± 0.000ᵃᵇ	2.39 ± 0.04ᵇ
BP6	14.44 ± 0.03ᵈ	5.00 ± 0.01ᵇ	0.419 ± 0.001ᵈ	3.02 ± 0.03ᵇ
BP7	24.15 ± 0.02ᵉ	5.10 ± 0.01ᵃᵇ	0.418 ± 0.000ᵈ	0.92 ± 0.01ᵃ
BP8	24.16 ± 0.01ᵉ	4.90 ± 0.01ᵇ	0.404 ± 0.001ᶜᵈ	2.79 ± 0.04ᵇ

*Note:* Values are expressed as mean ± standard deviation (*n* = 3). Different superscript letters within the same column indicate significant differences according to Tukey's test (*p* < 0.05).

### 3.5 Ash

The ash content in our samples ranged from a lower value of 0.922 ± 0.01 for samples harvested from Wazzan to a higher value of 4.635 ± 0.03 for those obtained from El Hajeb, with an average of 3.19 (Table 3). These results align with those reported by Asmae et al. (2021) where values ranged from 1.81% to 4.02, and those of (Aylanc et al. 2023) who reported an average of 3.3%. All studied samples comply with Brazilian and Argentinian regulations which authorized a maximum ash content of 6% (Straumite et al. 2022). Ash content of BP is crucial to its nutritional value. However, it can be significantly influenced by a various factor including soil characteristic, botanical origin, environmental conditions and methods of harvesting and manufacturing (Aylanc et al. 2023).

### 3.6 Minerals

The mineral composition analysis of the BP samples collected from different apiaries during spring 2023 (March–April) identified 10 elements (Table 4); Potassium K and calcium Ca were the predominant macroelements with (K) concentrations ranging from 741.82 ± 23.58 mg/kg (BP4) to 2116.99 ± 56.62 mg/kg (BP8) and Ca from 676.53 ± 16.37 mg/kg (BP6) to 2191.15 ± 13.06 mg/kg (BP3). One‐way ANOVA confirmed significant inter‐sample variation for both elements (K: F = 12.45. *p* < 0.01; Ca: F = 15.32. *p* < 0.001). Mg ranked third (255.65 ± 14.72 to 589.60 mg/kg), followed by phosphorus (P; 14.00 ± 1.79 to 126.43 ± 4.67 mg/kg), both showing significant variability (Mg: ANOVA F = 8.76. *p* < 0.05; P: F = 10.21. *p* < 0.01) (Table S1).

**TABLE 4:** Mineral elements content in BP samples (mg/kg).

Minerals	BP1	BP2	BP3	BP4	BP5	BP6	BP7	BP8
K	1746.9 ± 19.2ᵈ	953.4 ± 40.6ᵇ	1565.9 ± 60.9ᶜ	741.8 ± 23.6ᵃ	737.3 ± 443.3ᵃ	1478.7 ± 18.9ᶜ	1062.5 ± 60.1ᵇ	2117.0 ± 56.6ᵉ
Ca	1292.9 ± 11.9ᶜ	1555.5 ± 30.8ᵈ	2197.2 ± 13.1ᶠ	1182.2 ± 14.8ᵇ	714.4 ± 1.4ᵃ	676.5 ± 16.4ᵃ	1373.3 ± 9.8ᶜ	1893.5 ± 15.6ᵉ
Mg	255.7 ± 14.7ᵃ	415.3 ± 9.2ᶜ	589.1 ± 6.1ᵉ	318.8 ± 5.3ᵇ	257.3 ± 8.3ᵃ	313.2 ± 3.5ᵇ	352.3 ± 7.2ᵇ	574.6 ± 5.7ᵈᵉ
P	75.6 ± 2.6ᶜ	108.9 ± 3.1ᵈ	126.4 ± 4.7ᵉ	76.3 ± 46.1ᶜ	65.8 ± 6.9ᵇᶜ	14.0 ± 1.8ᵃ	57.0 ± 4.5ᵇ	19.9 ± 2.8ᵃ
Fe	19.6 ± 0.8ᵃ	104.4 ± 9.1ᵉ	57.9 ± 6.8ᶜ	16.1 ± 0.4ᵃ	16.5 ± 2.8ᵃ	14.8 ± 0.9ᵃ	98.5 ± 3.2ᵈ	60.7 ± 2.3ᵇ
Zn	5.8 ± 0.5ᵃ	34.0 ± 1.3ᶜ	21.9 ± 1.7ᵇ	14.8 ± 0.8ᵃᵇ	18.2 ± 0.9ᵇ	53.8 ± 2.0ᵈ	22.2 ± 1.8ᵇ	14.3 ± 1.2ᵃᵇ
Na	11.8 ± 2.0ᵃᵇ	11.2 ± 0.3ᵃᵇ	3.7 ± 0.5ᵃ	17.1 ± 1.9ᵇᶜ	10.1 ± 0.9ᵃᵇ	25.4 ± 1.9ᶜ	22.9 ± 2.0ᶜ	43.3 ± 1.4ᵈ
Se	1.08 ± 0.02ᶜ	0.61 ± 0.06ᵇ	0.73 ± 0.04ᵇᶜ	0.84 ± 0.05ᵇᶜ	0.21 ± 0.01ᵃ	1.66 ± 0.14ᵈ	1.26 ± 1.30ᶜᵈ	0.35 ± 0.01ᵃᵇ
Pb	ND	0.09 ± 0.02ᵃ	0.56 ± 0.04ᵇ	ND	ND	0.11 ± 0.01ᵃ	0.25 ± 0.02ᵃᵇ	ND
Cd	ND	ND	0.03 ± 0.00ᵃ	ND	0.02 ± 0.00ᵃ	ND	ND	ND

*Note:* Values are expressed as mean ± standard deviation (*n* = 3). Different superscript letters within the same row indicate significant differences according to Tukey's post hoc test (*p* < 0.05).

Abbreviation: ND, not detected.

These Macro element profiles align closely with Moroccan studies by Laaroussi et al. (2023) who reported K (1049.9–2972.6 mg/kg) and Ca (980.3–2343.6 mg/kg) as dominant in multifloral samples from various regions in Morocco with (Mg) averaging 527.9 mg/kg. Asmae et al. (2021) found comparable K ranges (485–4594 mg/kg) and Mg (69–793 mg/kg) in Moroccan BP samples attributing dominance to floral diversity and soil mineralization. However, our P levels (14–126 mg/kg) were notably lower than (Aylanc et al. 2023) for Portuguese samples (467 mg/100 g≈4670 mg/kg) where P predominated. Another Spanish study (71 samples) identifying P‐K as leading elements (Valverde et al. 2023). Turkish pollen showed higher K (1980–4099 mg/kg) but similar P variability confirming regional soil and botanical influences (Spulber et al. 2018).

Trace elements included iron (Fe; 14.76 ± 0.94 to 104.39 ± 9.04 mg/kg), zinc (Zn; 3.72 ± 0.52 to 43.28 ± 1.41 mg/kg), sodium (Na; 5.82 ± 0.45 to 53.77 ± 2.03 mg/kg) and selenium (Se; 0.21 ± 0.017 to 1.66 ± 0.14 mg/kg). These match Turkish profiles (Fe 47.3 mg/kg. Zn 19.9 mg/kg ranked Fe > Al > Zn) and Ethiopian studies with weak geographical correlations (Pearson's *r* < 0.3. *p* > 0.05) reflecting discrete edaphic sources rather than apiary proximity, such macro‐trace patterns support K, Ca, and Mg roles in electrolyte balance, bone health, and enzymatic functions consistent with global reviews (Oroian et al. 2022).

Cadmium (Cd) was detected at 0.035 mg/kg (BP3) and 0.029 mg/kg (BP5), while lead (Pb) reached 0.09 mg/kg (BP2), 0.56 mg/kg (BP3). All values met analytical quality criteria (LOQ: Cd 0.01 mg/kg Pb 0.05 mg/kg; recoveries 95%–102%. RSD < 5%) compared to Codex guidance (< 0.2 mg/kg Cd. < 0.5 mg/kg Pb) and EU Reg. 1881/2006 honey limits (Pb ≤ 0.1 mg/kg), all detected concentrations remained within the recommended limits. The detected levels were also lower than those reported in Turkish studies (Pb up to 1.2 mg/kg) or Italian apiary maxima. For a 10 g daily intake, exposure would equal 0.0129 μg Pb/kg BW/day (BP2) and 0.0041 μg Cd/kg BW/day (BP5) for a 70 kg adult, remaining below the EFSA BMDL01 for Pb (0.63 μg/kg BW/day) and the PTWI for Cd (2.5 μg/kg BW/week), although continued monitoring is advisable, particularly for children.

A moderate Cd‐Pb correlation (*r* = 0.560. *p* < 0.05) mirrors Abruzzo findings (Blanc et al. 2021) suggesting shared anthropogenic sources (fertilizers, traffic, industry) near apiaries. High variability (CV = 45%–62%) stems from floral origins, soil pH, pollution and nectar dilution limitations of *n* = 8 spring samples. These results position BP as an environmental bioindicator, warranting multi‐season studies, botanical validation and soil correlations. As recommended in recent Ethiopian and Kazakh pollen research (Gardana et al. 2018).

### 3.7 Carbohydrates

Carbohydrates derived from flower nectar are the major component of BP accounting for approximately 70% of its dry weight (Thakur and Nanda 2020). They are the main source of nutrition for honeybees (Qiao et al. 2023). The carbohydrate content of the samples ranged from 17.20 ± 0.22 g/100 g (dw) in BP7 harvested from Wazzane, to 28.87 ± 0.08 g/100 g dw in BP2 harvested from El Hajeb with an average of 20.93 ± 0.107 g/100 g dw (Table 5). These results are in accordance with the range reported by Campos et al. (2008) who suggested values between 13 and 55 g/100 g dw. Our results are lower than those reported by Laaroussi et al. (2023) who reported a mean carbohydrate value of 23.26 ± 2.26 g/100 g dw with a range of 18.52 ± 0.19 g/100 g dw to 46.44 ± 0.55 g/100 g dw. This variation is probably due to the many factors that influence the composition of BP such as geographical location, climate, soil type, plant origin, growing conditions, harvesting season, bee species, beekeeping practices and storage techniques. In a study of 69 BP samples collected from two regions in northeastern Portugal, high carbohydrate levels were observed in Vimioso (66.60% ± 3.52%) and Mogadouro (63.35% ± 2.79%) (Pascoal et al. 2023). Another study carried out in Serbia reported carbohydrate contents ranging from a minimum of 64.42 g/100 g to a maximum of 81.84 g/100 g, with an average of 75.51 ± 4.25 g/100 g (Kostić et al. 2015). These values are significantly higher than those observed in our study. The higher carbohydrate content can be attributed to the incorporation of honey or nectar during pelleting. However, the level of carbohydrates is also greatly influenced by factors such as plant origin, growth and harvesting conditions (Thakur and Nanda 2020).

**TABLE 5:** Physicochemical composition and energetic value of BP samples.

Bee pollen	Soluble proteins (g BSA/100 g dw)	Total proteins (g/100 g dw)	Lipids (g/100 g dw)	Soluble carbohydrates (g GlcE/100 g dw)	Energy (kcal/100 g dw)
BP1	15.42 ± 0.02ᵇᶜ	18.90 ± 0.01ᶜ	2.44 ± 0.43ᵃᵇ	18.97 ± 0.06ᵇ	173.44 ± 1.02ᵇ
BP2	13.64 ± 0.05ᵃ	15.89 ± 0.02ᵃ	2.93 ± 0.51ᵇ	28.87 ± 0.08ᵈ	205.41 ± 1.33ᵈ
BP3	13.45 ± 0.08ᵃ	14.55 ± 0.05ᵃ	2.85 ± 0.04ᵇ	18.25 ± 0.02ᵇ	156.85 ± 0.92ᵃ
BP4	15.37 ± 0.06ᵇᶜ	17.67 ± 0.01ᵇ	2.41 ± 0.50ᵃᵇ	27.19 ± 0.06ᶜ	201.13 ± 1.01ᶜᵈ
BP5	15.21 ± 0.09ᵇ	15.65 ± 0.02ᵃ	1.92 ± 0.31ᵃ	20.09 ± 0.09ᵇ	160.24 ± 2.00ᵃ
BP6	16.08 ± 0.03ᶜ	18.31 ± 0.01ᶜ	3.03 ± 0.03ᵇ	17.77 ± 0.14ᵃᵇ	171.59 ± 1.07ᵇ
BP7	16.07 ± 0.05ᶜ	17.27 ± 0.03ᵇ	2.56 ± 0.56ᵃᵇ	17.20 ± 0.22ᵃ	160.92 ± 1.02ᵃ
BP8	16.08 ± 0.06ᶜ	19.56 ± 0.06ᵈ	2.94 ± 0.95ᵇ	19.12 ± 0.19ᵇ	181.18 ± 2.01ᵇᶜ

*Note:* Values are expressed as mean ± standard deviation (*n* = 3). Different superscript letters within the same column indicate significant differences according to Tukey's post hoc test (*p* < 0.05).

### 3.8 Lipids

Lipids are the third most abundant component in BP following carbohydrates and proteins. Among the lipids which are present in pollen in an amount of about 5.1%, the most important are the essential fatty acids (Qiao et al. 2023). The lipid content in the examined samples ranged from a minimum of 1.92 ± 0.31 g/100 g in BP5, harvested from Sidi Slimane to a maximum of 3.03 ± 0.31 g/100 g in BP6 harvested from Boufkrane, with an average value of 2.635 ± 0.41 g/100 g (Table 5). These findings align with the values proposed by Campos et al. (2008) which fall within the range of 1 g/100 g to 13 g/100 g. Our results are slightly lower than those reported by Kostić et al. (2015) which showed values ranging from 1.31 g/100 g to 6.78 g/100 g, with an average of 2.66 ± 1.23 g/100 g.

### 3.9 Proteins

Proteins are the largest component in pollen after carbohydrates and fulfill the honeybee nutritional requirements (Thakur and Nanda 2020). The protein content in this product is frequently regarded as a quality index of its nutritional value.

#### 3.9.1 Soluble Proteins

Soluble proteins varied between 13.45% in BP3 obtained from Sidi Kassem and 16.08% in BP7 from Ouazzane (Table 5). These results are less than those reported by Laaroussi et al. (2023) showing values ranging from 16.63 ± 3.66 gBSA/100 g dw to 31.17 ± 3.81 (9). The protein content in Polish pollen amounts to 20.7%, whereas, it is 17% in Spanish pollen, 20.7% in Korean pollen and 23.7% in a Chinese one. In Brazilian pollen the protein content is 21.4% (Rzepecka‐Stojko et al. 2015).

#### 3.9.2 Total Proteins

The total protein content of the BP samples showed considerable variation between the different types. The lowest total protein content was observed in BP3 from sidi kacem with a value of 14.55 g/100 g (dw). The highest values, with a total protein content of 19.56 g/100 g (dw), were observed in BP8 (Table 5).

These results are in line with previous studies such as those of (Kostić et al. 2015) who reported protein contents ranging from 14.81 to 37.25 g/100 g in Serbian BP samples and (Laaroussi et al. 2023) who found 19.18 g/100 g (dw) and 16.63 ± 3.66 g BSA/100 g (dw). Similarly, Bogdanov noted a typical protein content range of 10–40 g/100 g (dw) for BP (Bogdanov 2016).

### 3.10 Energy Value

The energy values of the analyzed BP samples showed considerable variation. The lowest energy value was observed in BP3 with 156.85 kcal/100 g (dw), while the highest value was recorded in BP2 with 205.41 kcal/100 g (dw) (Table 5). These values are significantly lower than those reported by Kostić et al. (2015) for Serbian BP samples which ranged from 351 to 396 kcal/100 g (dw) and (Laaroussi et al. 2023) (196.3 ± 1.0 kcal/100 g (dw) and 247.1 ± 2.2 kcal/100 g (dw)). The discrepancies in energy content can be attributed to differences in the chemical composition of BP which are influenced by botanical origin environmental conditions and regional variations.

### 3.11 Antioxidant Activity

Our data highlight significant diversity in phenolic compounds among the samples. This variability is evident in the recorded values for BP3 and BP5 which range from 4.19 to 17.39 milligrams of Gallic Acid Equivalent (GAE) per gram of (dw). Respectively (Table 6). These values are generally consistent with previous reports on Moroccan BP. A study on Moroccan BP reported TPC values ranging from 12.40 to 16.85 mg GAE/g (Laaroussi et al. 2023), which fall within the upper range of our findings. Similarly, another investigation on Moroccan monofloral BP found a TPC range of 8.07–32.38 mg GAE/g, encompassing both lower and higher values than those obtained in the present study (Asmae et al. 2021). Additionally, the present results are comparable to those reported for BP from other geographical origins, including Brazilian samples (6.50 to 29.20 mg GAE/g) and Indian samples (9.79 to 35.63 mg GAE/g) (De‐Melo et al. 2018; Thakur and Nanda 2021). The analysis of flavonoid content also revealed differences among the samples. While PB1 had a minimal flavonoid content of approximately 0.94 milligrams of quercetin equivalent (QE) per gram of (dw). PB5 showed a much higher concentration, with about 3.18 milligrams QE/100g. These findings emphasize the wide range of flavonoid concentrations present in the BP samples analyzed. Our results were in the same range as those reported in a study conducted by Laaroussi et al. (2023) for Moroccan BP (1.51 to 4.57) and (Boulfous et al. 2025) for Algerian BP (1.48 to 5.57 mg QE/g) but lower than those reported for Romanian BP (4.93 to 20.45 mg QE/g) (Oroian et al. 2022), Indian BP (9.72 to 15.61) (Thakur and Nanda 2021) and Brazilian pollen, where the TFC was 0.30–17.50 mg QE/g of pollen extract (De‐Melo et al. 2018). The observed differences can be attributed to several factors, with botanical origin being the main factor, followed by geographical and climatic variations (Kolayli et al. 2023).

**TABLE 6:** Antioxidant content of the ethanolic extract of BP.

BP	TPC (mg GAE/g dw)	TFC (mg QE/g dw)	DPPH IC_50_ (mg/mL)	FRAP (μmol Fe^2+^/g)	ABTS IC_50_ (mg/mL)
BP1	5.01 ± 0.57ᵃ	0.94 ± 0.26ᵃ	0.64 ± 0.08ᵈ	94.42 ± 0.05ᵃ	0.28 ± 0.02ᵈ
BP2	8.81 ± 0.22ᵇ	2.02 ± 0.20ᵇ	0.41 ± 0.06ᵇᶜ	146.92 ± 0.05ᵈ	0.12 ± 0.01ᵃ
BP3	4.19 ± 0.24ᵃ	2.28 ± 0.09ᵇᶜ	0.53 ± 0.07ᶜᵈ	87.33 ± 0.05ᵃ	0.24 ± 0.02ᶜ
BP4	14.98 ± 3.63ᶜ	2.49 ± 0.10ᵇᶜ	0.45 ± 0.06ᵇᶜ	119.92 ± 0.07ᵇ	0.14 ± 0.01ᵃᵇ
BP5	17.39 ± 1.22ᶜ	3.18 ± 0.01ᵈ	0.40 ± 0.06ᵇ	138.63 ± 0.10ᶜ	0.16 ± 0.01ᵇ
BP6	11.48 ± 0.33ᶜ	2.31 ± 0.66ᵇᶜ	0.43 ± 0.06ᵇᶜ	131.58 ± 0.14ᶜ	0.21 ± 0.02ᶜ
BP7	14.62 ± 3.83ᶜ	2.03 ± 0.74ᵇ	0.42 ± 0.06ᵇᶜ	98.13 ± 0.13ᵃ	0.20 ± 0.02ᶜ
BP8	13.46 ± 0.94ᶜ	2.09 ± 0.65ᵇ	0.28 ± 0.00ᵃ	113.62 ± 0.09ᵇ	0.13 ± 0.01ᵃᵇ

*Note:* Values are expressed as mean ± standard deviation (*n* = 3). Different superscript letters within the same column indicate significant differences according to Tukey's post hoc test (*p* < 0.05).

To evaluate antioxidant activity, three different and complementary assays FRAP, DPPH and ABTS were used to assess various antioxidant mechanisms. The significant differences in the chemical composition and concentrations of phenolic and non‐phenolic antioxidant molecules along with the complex chemistry of the assays contribute to variations in the antioxidant activity results. As shown in (Table 6). The samples (PB1‐PB8) exhibit distinct antioxidant activities across the different methods employed.

The 2.2‐diphenyl‐1‐picrylhydrazyl (DPPH) radical scavenging assay is a common method of evaluating antioxidant activity. As shown in (Table 4), the amount of BP needed to inhibit 50% of the DPPH^•^ radical varied significantly among the samples tested. The BP sample from the Sebt Jahjouh area (PB2) exhibited the lowest antiradical activity with an IC_50_ value of 0.64 mg/mL. In contrast, the sample from Douar Lafdoul (PB8) demonstrated the highest DPPH scavenging capacity, with an IC_50_ of 0.28 mg/mL. These differences highlight the important role of floral origin and the pedo‐climatic conditions of the harvesting sites, together with differences in the phenolic and non‐phenolic antioxidant profiles of the samples.

Although all samples showed varying levels of antiradical activity, their IC_50_ values were lower than that of Trolox which has an IC_50_ of 10.81 ± 0.1 μg/mL indicating higher antiradical activities. Our findings align closely with those from a study in Morocco which reported a mean IC_50_ of 0.39 mg/mL. However, they were significantly higher than those reported in Brazil (0.86 mg/mL), Chilie (2.36 mg/mL), China (1.28 mg/mL), Portugal (3 mg/mL), and Spain (2.98 mg/mL) (Duarte et al. 2018; El Ghouizi et al. 2020; Feas et al. 2012; Pascoal et al. 2014).

Among the samples tested for ABTS radical scavenging capacity, the one from the Sebt Jahjouh region (PB2) demonstrated the highest activity, with an IC_50_ value of 0.12 mg/mL. In contrast, the sample from the Jorf El Malha area (PB1) showed the lowest inhibition percentage with an IC_50_ of 0.28 mg/mL. These results indicate a strong antiradical activity particularly when compared to Trolox which has an IC_50_ of 23.15 μg/mL. Moreover, the observed scavenging capacities significantly exceed those reported in a similar study conducted in Greece which found an IC_50_ of 56.2 μg/mL (Atsalakis et al. 2017). The variation in antioxidant activity observed across the analyzed BP samples is consistent with findings reported in the literature. The unexpected negative correlations between total phenolic content (TPC) and antioxidant activities (DPPH, ABTS) (*r* = −0.633 to −0.692) suggest that factors beyond total phenol concentration are driving the antioxidant capacity. This may include the specific profile of phenolic compounds (e.g., some flavonoids are more potent than phenolic acids), the presence of non‐phenolic antioxidants such as vitamins or peptides, or potential antagonistic effects between different bioactive compounds in the pollen matrix. The samples showed considerable differences in their antioxidant capacities likely due to their unique phytochemical compositions and the presence of various antioxidant compounds. These findings highlight the potential of honey as a bioactive functional food and suggest that its inclusion in the daily diet may provide health benefits.

Comparison of the FRAP assay results with those reported by other researchers showed that the obtained values (87.33–146.92 μmol Fe^2+^/g dw) were similar to those reported by Barbieri et al. (2020) which ranged from 14.77 to 190.27 μmol Fe^2+^/g, and higher than those reported by Saral et al. (2019) who analyzed Turkish BP samples and found values ranging from 8.69 to 84.89 μmol Fe^2+^/g.

It has been reported that BP possesses antioxidant properties that greatly depend on various factors, including bee species, plant source, environmental conditions, production methods, and storage conditions. In addition, the complex and variable composition of this bee product, together with the diversity of methods used to determine its antioxidant activities, results in a wide range of findings reported in numerous studies (Kolayli et al. 2024).

### 3.12 In Vitro Digestive Enzymes' Inhibitory Activity

Diabetes is a metabolic and degenerative disease with significant long‐term consequences. It is a leading cause of numerous complications, including blindness, heart attacks, lower limb amputations and kidney failure. Type 2 diabetes is the most common form. Inhibition of pancreatic amylase can regulate postprandial blood glucose levels in diabetics (Araújo et al. 2017). α‐Amylase and α‐glucosidase are crucial enzymes in the digestion of dietary carbohydrates and the subsequent release of glucose (Laaroussi et al. 2023). The IC_50_ values for the inhibition of both α‐amylase and α‐glucosidase by various BP samples (BP1‐BP8) show significant variability in their in vitro antihyperglycemic effect. For α‐amylase, IC_50_ values range from 263.40 ± 53.65 μg/mL (BP5) to 953.66 ± 3.12 μg/mL (BP1) with both values higher than those reported by acarbose (IC_50_ = 35.42 ± 1.0 μg/mL) (Table 7). Current results are comparable to those reported by Laaroussi et al. (2023), where IC_50_ values of seven Moroccan BP samples ranged from 171.32 ± 3.13 μg/mL to 979.26 ± 7.34 μg/mL. It is most important to mention that BP5 showed the highest amount of TPC and TFC. in contrast, the BP1 displayed the lowest TPC and TFC values, which support the hypothesis that phenolic and flavonoid constituents significantly contribute to the α‐amylase the α‐amylase inhibition mechanism. Nine types of BP were collected by beekeepers in Brazil among these, the highest IC_50_ value was 1015.19 μg/mL. observed in the sample from Neopolis, while the lowest IC_50_ value recorded was 16.44 μg/mL (Araújo et al. 2017) For α‐glucosidase, as compared to standard drug. Acarbose, which displayed an IC_50_ value of 11.000 ± 1.0 μg/mL investigated BP extracts reported higher IC_50_ values ranged from 145.50 ± 22.12 μg/mL (BP6) to 670.26 ± 11.67 μg/mL (BP1). This indicates that BP5 and BP6 are the most potent inhibitors of α‐amylase and α‐glucosidase, respectively, suggesting they have the highest potential to reduce postprandial glucose (PPG) by limiting the digestion and thereby delaying the intestinal absorption of glucose. These findings align with those of (Laaroussi et al. 2023) who reported IC_50_ values ranging from 107.99 ± 5.16 μg/mL to 661.05 ± 18.03 μg/mL.

**TABLE 7:** α‐amylase and α‐glucosidase contents in BP samples.

Enzyme	BP1	BP2	BP3	BP4	BP5	BP6	BP7	BP8
α‐Amylase	953.7 ± 3.1ᵉ	538.9 ± 14.0ᶜ	512.2 ± 9.5ᶜ	318.0 ± 8.4ᵇ	263.4 ± 53.7ᵃ	440.9 ± 50.0ᶜ	266.0 ± 6.0ᵃ	768.2 ± 40.1ᵈ
α‐Glucosidase	670.3 ± 11.7ᵉ	553.6 ± 8.5ᵈ	256.6 ± 10.1ᵇ	241.1 ± 6.4ᵇ	189.0 ± 36.4ᵃᵇ	145.5 ± 22.1ᵃ	230.5 ± 14.5ᵇ	290.8 ± 12.9ᶜ

*Note:* Values are expressed as mean ± standard deviation (*n* = 3). Different superscript letters within the same row indicate significant differences according to Tukey's post hoc test (*p* < 0.05).

The effectiveness of α‐amylase and α‐glucosidase inhibitors in managing diabetes lies in their ability to delay the breakdown of complex carbohydrates into glucose, thus reducing blood glucose spikes after meals. The substantial variability in IC_50_ values among the BP samples suggests that their inhibitory potency is closely linked to their specific phytochemical composition which may vary according to the botanical and geographical origins of the pollen. This observation is consistent with previous studies showing wide‐ranging IC_50_ values influenced by antioxidant activity and flavonoid content (Daudu 2019; Laaroussi et al. 2023), Particularly, samples BP5 and BP6 are notable for their dual inhibitory effects on both α‐amylase and α‐glucosidase. This dual action makes them potentially powerful natural agents for regulating postprandial glucose (PPG), which is recognized as a promoting target for the prevention and management of diabetes, particularly type 2 diabetes mellitus (T2DM), in which postprandial glycemic control is a major therapeutic objective. The differences among the samples highlight the importance of standardizing BP preparations to ensure consistent therapeutic efficacy. Specific phenolic compounds, particularly flavonoids like quercetin and luteolin, play a crucial role as they interact with the active sites of α‐amylase and α‐glucosidase through hydrogen bonding and π‐system interactions (Şöhretoğlu and Sari 2020; Tadera et al. 2006). Additionally, polyphenols can limit α‐amylase or α‐glucosidase activity and reduce the formation of reactive derivatives in non‐enzymatic glucose‐protein condensation reactions (Daudu 2019).

### 3.13 Phenolic Compounds Identification

In the BP1 sample, seven compounds were detected with three being positively identified: Naringin, Gallic acid and 3‐hydroxybenzoic acid. Naringin a flavonoid glycoside is known for its antioxidant anti‐inflammatory and cardioprotective properties making it valuable in therapeutic applications. Gallic acid a well‐known phenolic acid exhibits potent antioxidant, antimicrobial, and anticancer activities. Meanwhile 3‐hydroxybenzoic acid another phenolic compound has been reported to possess antioxidant and anti‐inflammatory activities. In the BP2 sample, eight compounds were detected with gallic acid being the only identified compound, its presence highlights the potential antioxidant and antimicrobial properties of this sample. Similarly, both BP3 and BP4 samples revealed six compounds each. In BP3, Naringin and Gallic acid were identified reinforcing the potential antioxidant and anti‐inflammatory benefits of this sample. In contrast, BP4 contained one identified compound (p‐coumaric acid) a phenolic compound known for its antioxidant, anti‐inflammatory, and antimicrobial effects. The BP5 sample exhibited the lowest compound diversity with only four compounds detected. Among these, Rutin was the sole identified compound. Rutin a flavonoid has been extensively studied for its antioxidant anti‐inflammatory and vasoprotective activities as well as its role in improving cardiovascular health. This pattern was also observed in the BP6 sample which detected rutin as the only identifiable compound suggesting similar biological activities for these two samples. In contrast. BP7 demonstrated the greatest compound richness with 20 compounds detected. Among the identified compounds were gallic acid, vanillic acid, and rutin. Vanillic acid a phenolic acid has been shown to possess antioxidant, anti‐inflammatory, and anti‐cancer properties. The combination of these compounds in BP7 suggests a strong potential for antioxidant and anti‐inflammatory effects. Lastly, in the BP8 sample 14 compounds were detected with three major compounds; Gallic acid, vanillic acid, and rutin being identified indicating a similar biological activity profile to BP7 (Figure 3). A preliminary cross‐referencing of phenolic profiles suggests potential links; for instance, the presence of Naringin in BP1 and BP3 aligns with their reported *Citrus sinensus* content a known source of this flavonoid. Similarly, Rutin was prevalent in samples (BP5‐BP8) containing plant species from the *Brassicaceae* (e.g., *Sinapis arvensis* ) and *Papaveraceae* ( *Papaver rhoeas* ) families, which are often rich in this compound. These observations highlight the potential of phenolic markers for botanical tracing and warrant targeted investigation in future studies.

**FIGURE 3:**
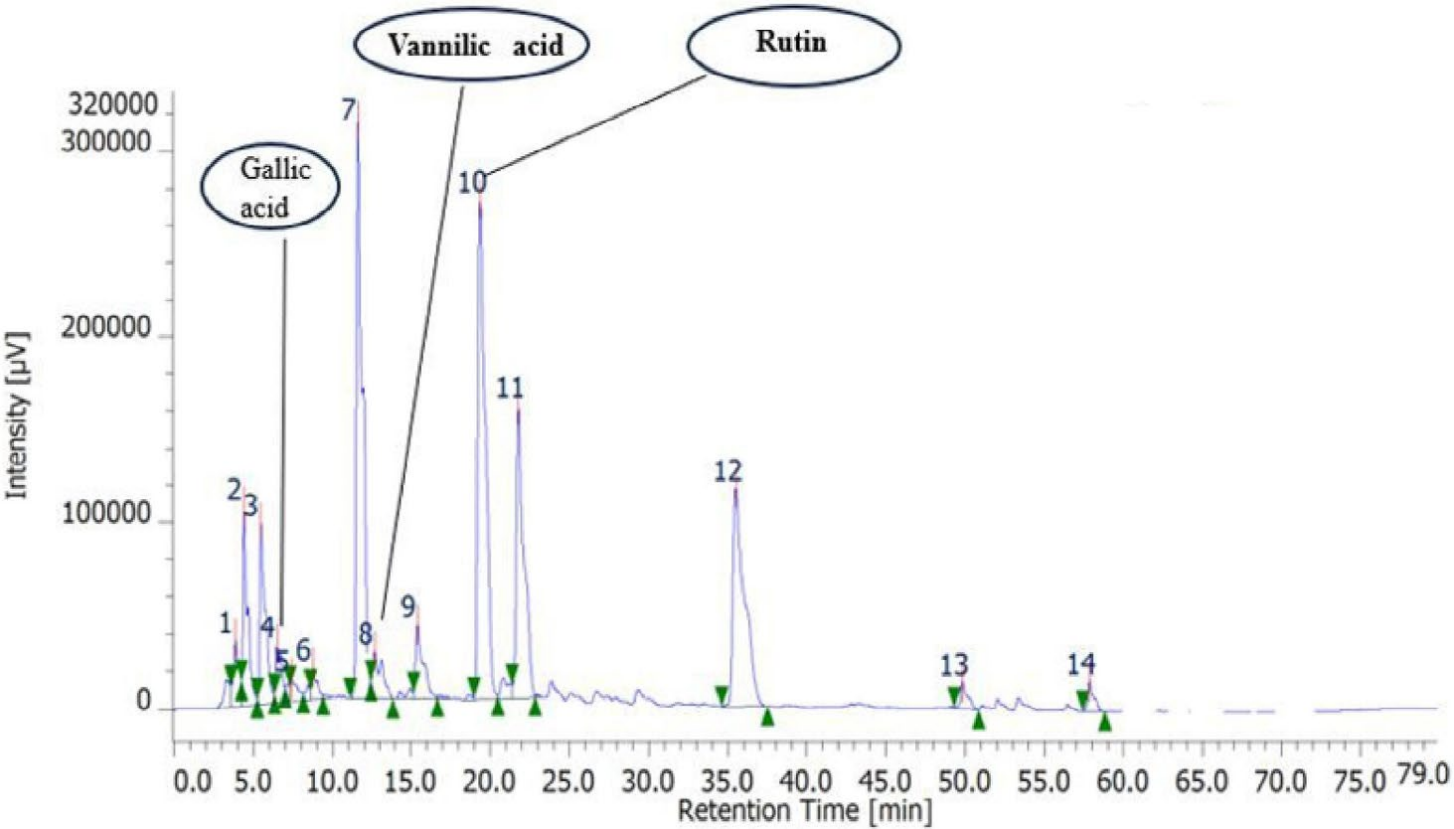
HPLC chromatographic profile at 280 nm for BP8 phenolic extract.

### 3.14 PCA Analysis

The correlation analysis reveals complex relationships among antioxidant, nutritional, and mineral components in Moroccan BP. The strong positive correlation between ABTS and DPPH (*r* = 0.836) confirms consistency between these antioxidant assays, while their negative correlations with polyphenols (*r* = −0.633 to −0.692) present an unexpected finding that warrants further investigation as this contradicts typical expectations of polyphenols enhancing antioxidant activity.

Nutritional components show significant interrelations: flavonoids demonstrate strong positive correlation with polyphenols (*r* = 0.669) but negative association with alpha‐amylase (*r* = −0.796) suggesting potential enzymatic inhibition. Carbohydrates maintain their expected strong negative correlation with water activity (*r* = −0.901) reflecting their hygroscopic nature. The protein component shows particularly strong negative correlation with phosphorus (*r* = −0.909) likely indicating important biochemical interactions in pollen composition.

Mineral analysis reveals expected patterns with calcium and magnesium showing particularly strong co‐occurrence (*r* = 0.881) consistent with their biological synergy. Heavy metals lead and cadmium maintain a moderate correlation (*r* = 0.560) possibly indicating common environmental sources of contamination. Enzymatic activities (alpha‐amylase and alpha‐glucosidase) show strong positive correlation (*r* = 0.873) suggesting coordinated functionality.

## 4 Conclusions

This study provides an integrated characterization of 8 Moroccan BP samples collected from various geographic regions, encompassing their nutritional composition, mineral profile, phenolic content, antioxidant capacity, and enzyme inhibitory activities. Moroccan BP is a nutrient‐dense product rich in proteins, potassium, calcium, and phenolic compounds, exhibiting moderate antioxidant activity and significant α‐amylase and α‐glucosidase inhibitory potential. BP is a natural product appreciated worldwide for its remarkable nutritional value and is increasingly recognized as an important functional food ingredient. Thanks to its rich bioactive composition, Moroccan BP shows promising contributions to oxidative stress modulation and postprandial glycemic control, highlighting its relevance in the context of growing consumer demand for natural health‐promoting foods. Today's consumers increasingly seek foods with demonstrated functional properties, and BP, with its diverse phenolic profile and enzyme inhibitory potential, represents a valuable candidate for functional food and nutraceutical applications. The present work establishes a valuable compositional baseline for Moroccan BP and contributes to the scientific foundation required for its quality standardization and sustainable valorization. Nevertheless, future research should focus on multi‐season and multi‐regional studies to better capture compositional variability, while incorporating standardized chromatic classification alongside melissopalynological analysis to further refine the characterization of BP heterogeneity. In vivo and clinical trials are also needed to validate the observed bioactivities, and advanced metabolomic profiling using HPLC‐MS/MS should be employed to identify individual phenolic compounds and establish structure–activity relationships. Bioaccessibility studies and molecular docking approaches are likewise warranted to support the evidence‐based integration of Moroccan BP into functional food strategies.

## Author Contributions

**Fedoua Diai:** conceptualization, formal analysis, funding acquisition, investigation, methodology, writing – original draft, writing – review and editing. **Toufik Bouddine:** conceptualization, investigation, methodology, supervision, writing – original draft, writing – review and editing. **Mohamed Reda Kachmar:** formal analysis, software, validation, writing – review and editing. **Morad Kaddouri:** visualization, writing – review and editing, project administration, writing – original draft. **Charaf Eddine Kassimi:** formal analysis, investigation, methodology, writing – original draft. **Ibtissame Guirrou:** formal analysis, methodology, writing – original draft. **Hassan Laaroussi:** methodology, formal analysis, writing – original draft. **Mohammed Al‐zharani:** visualization, funding acquisition, writing – review and editing. **Fahd A. Nasr:** visualization, validation, resources, funding acquisition. **Ashraf Ahmed Qurtam:** writing – review and editing, resources, funding acquisition. **Lhoussain Hajji:** conceptualization, data curation, resources, visualization, writing – review & editing, supervision, project administration. **Said Chakir:** writing – review and editing, visualization, project administration, supervision.

## Funding

This work was supported and funded by the Deanship of Scientific Research at Imam Mohammad Ibn Saud Islamic University (IMSIU) (grant number IMSIU‐DDRSP2601).

## Ethics Statement

The authors have nothing to report.

## Conflicts of Interest

The authors declare no conflicts of interest.

## Supporting information


**Table S1:** One‐way ANOVA results for physicochemical parameters, bioactive compounds, and enzyme inhibitory activities of Moroccan bee pollen samples (BP1–BP8). Degrees of freedom (df) are expressed as (between groups, within groups).
**Figure S1:** Calibration curve for total phenolic content (TPC) using gallic acid as standard.
**Figure S2:** Calibration curve for total flavonoid content (TFC) using quercetin as standard.
**Figure S3:** Principal component analysis (PCA) of Moroccan bee pollen samples. (A) Loading plot showing the contribution of each variable to PC1 and PC2. (B) Score plot showing the distribution of samples (BP1–BP8). (C) Scree plot showing the eigenvalues and variance explained by each principal component.

## Data Availability

The data that support the findings of this study are available from the corresponding author upon reasonable request.
