# Supplementary material for: Multi‐Regional Characterization of Moroccan Bee Pollen: Nutritional, Bioactive, and Functional Properties
Source: Food Sci Nutr. 2026 Oct 7;14(10):e72174. doi: 10.1002/fsn3.72174 (PMC13643948; doi:10.1002/fsn3.72174)
Supplement: Supplementary file 1 [file FSN3-14-e72174-s001.docx]

**Table S1 –** One‑way ANOVA results for physicochemical parameters, bioactive compounds, and enzyme inhibitory activities of Moroccan bee pollen samples (BP1–BP8). Degrees of freedom (df) are expressed as (between groups, within groups).

| Parameter | F-value | df | p-value |
| --- | --- | --- | --- |
| Moisture | 18.45 | (7, 16) | < 0.001 |
| pH | 5.32 | (7, 16) | 0.002 |
| Aw | 12.76 | (7, 16) | < 0.001 |
| Ash | 9.87 | (7, 16) | < 0.001 |
| TPC | 12.87 | (7, 16) | < 0.001 |
| TFC | 10.45 | (7, 16) | < 0.001 |
| DPPH IC50 | 15.32 | (7, 16) | < 0.001 |
| ABTS IC50 | 18.21 | (7, 16) | < 0.001 |
| FRAP | 22.45 | (7, 16) | < 0.001 |
| α‑amylase | 28.76 | (7, 16) | < 0.001 |
| α‑glucosidase | 31.22 | (7, 16) | < 0.001 |

| Gallic acid concentration | Absorbance |
| --- | --- |
| 0.025 | 2.08967 |
| 0.0125 | 1.11 |
| 0.00625 | 0.614667 |
| 0.003125 | 0.421 |
| 0.0015625 | 0.301 |
| 0.00078125 | 0.122 |

**Figure S1 –** Calibration curve for total phenolic content (TPC) using gallic acid as standard.

| Quercetin concentration | Absobance |
| --- | --- |
| 0.035 | 2.08967 |
| 0.0175 | 1.11 |
| 0.00875 | 0.614667 |

**Figure S2 –** Calibration curve for total flavonoid content (TFC) using quercetin as standard.

**A :**

**B :**

**C:**

**Figure S3 –** Principal component analysis (PCA) of Moroccan bee pollen samples. (A) Loading plot showing the contribution of each variable to PC1 and PC2. (B) Score plot showing the distribution of samples (BP1–BP8). (C) Scree plot showing the eigenvalues and variance explained by each principal component.
